# The visual impediment of cranial ornamentation in male *Chrysolophus* pheasants

**DOI:** 10.1098/rsbl.2025.0405

**Published:** 2025-11-26

**Authors:** Alexandra E. R. Lamond, Simon Potier, Laurent Fontaine, Graham R. Martin, Steven J. Portugal

**Affiliations:** ^1^ Department of Biological Sciences, Royal Holloway University of London, Egham, Surrey TW20 0EX, UK; ^2^ Simon Potier Expert Scientifique, 1 rue du vivier, Foucrainville 27220, France; ^3^ Les Ailes de l’Urga, 72 rue de la vieille route, Marcilly la Campagne 27320, France; ^4^ Président de la WPA France, 36 rue du quartier neuf, Portel des Corbières 11490, France; ^5^ School of Biosciences, The University of Birmingham, Edgbaston BH15 2TT, UK; ^6^ Department of Biological Sciences, The University of Oxford, Oxford OX1 3SZ, UK; ^7^ Bird Group, The Natural History Museum Tring, Akeman Street, Tring HP23 6AP, UK

**Keywords:** binocularity, Galliformes, moult, Phasianidae, sensory ecology, vision

## Abstract

Sexually selected traits such as feather ornamentation of male birds can act as an impediment to movement and predator detection. Here, we report a previously undocumented example of an impediment derived from a sexually selected trait: the cranial feather ornamentation in male *Chrysolophus* pheasants restricting their visual field. Visual fields define the space around an animal from which visual information can be retrieved. Out of the 300 bird species studied to date, there have been no significant differences reported in the visual fields between sexes. Our findings reveal that the cranial feathers of male golden (*C. pictus*) and Lady Amherst’s (*C. amherstiae*) pheasants significantly restrict their visual field relative to females and may impede their ability to gather information from the world about them. This effect is most extreme in the vertical extent, where a 30° and 40° difference is evident between the sexes of golden and Lady Amherst’s pheasants, respectively. The two *Chrysolophus* pheasant species are the first species studied to show a difference in visual fields between sexes; this difference was absent in two closely related species also measured in this study, silver pheasants (*Lophura nycthemera*) and green pheasants (*Phasianus versicolor*).

## 1. Introduction

The visual field plays a pivotal role in any visual system, defining the three-dimensional space from which an organism gathers visual information at any given moment [1,2]. Variations in eye size and optical structure shape the visual fields of each eye, and the way in which eyes are placed in the skull determines the overall dimensions of the visual field of an animal, including the extent and positions of the blind areas and binocular vision [3–5]. Among birds it has been shown that key elements of the visual field (monocular, binocular regions and blind regions) are closely associated with essential behaviours like foraging, caring for offspring, detecting predators and tracking other individuals [2,6–8]. Differences in visual field structure are also apparent in closely related species that occupy distinct ecological niches for acquiring food. For example, distinctions can be seen among ibises and spoonbills (Threskiornithidae [9], ducks, geese and swans (Anatidae [10]), finches (Emberizidae [11]) and birds of prey (Accipitriformes and Strigidae [12,13]). Within these groups, evidence suggests that foraging strategies, rather than evolutionary relationships, have a greater influence on visual field characteristics [10,13]. The binocular field—the area where monocular fields overlap—is of particular interest. The topography of the binocular field differs across species, reflecting the need to control the position of the bill (or talons) during particular foraging behaviours [14,15] or when feeding chicks [2]. Binocular vision allows precise control over the direction in which the bill (or feet) moves, as well as the timing of its contact with a target [16]. While foraging method, requirements for nest construction, and chick feeding are known to drive the evolution of visual fields [2,5], there is no evidence of sex differences in the visual fields of any bird species [8]. However, a hitherto unconsidered influence on visual field characteristics are secondary sexual characteristics such as ceres, casques and exaggerated cranial feathers. These structures could intrude into the visual fields and, because of their sexual dimorphism, give rise to sex differences in visual field topography.

**Figure 1.**
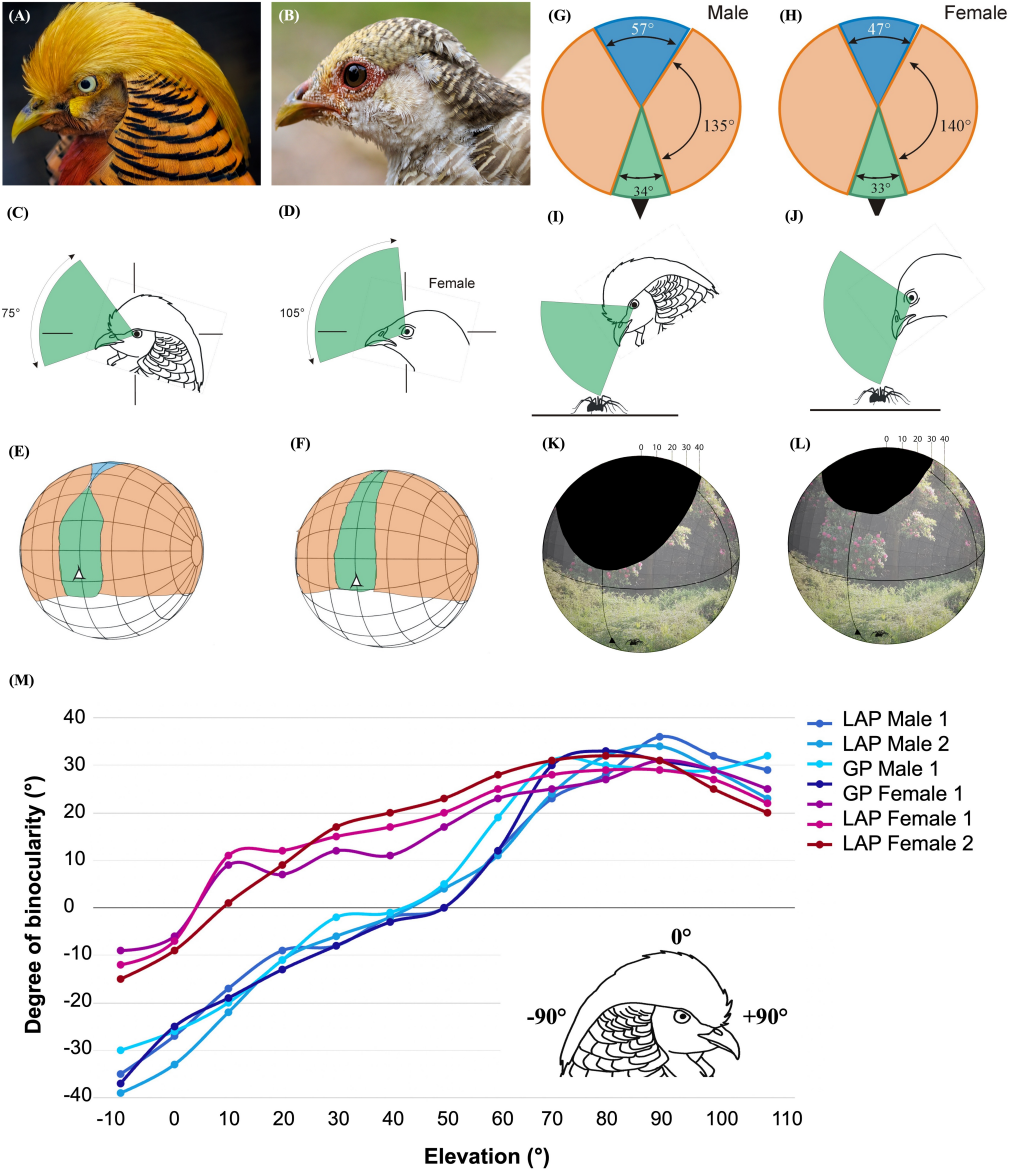
Visual field configurations in golden pheasants (*Chrysolophus pictus*, GP) and Lady Amherst’s pheasants (*C. amherstiae*, LAP) are illustrated. Panels (A) and (B) depict male and female golden pheasants, while panels (C) and (D) show vertical cross-sections through the binocular fields in the mid-sagittal plane of the head. The head drawings represent typical resting postures for each species, based on photographs of birds observed in aviaries. Panels (E) and (F) provide three-dimensional views of orthographic projections outlining the retinal field boundaries of both eyes, together with the eye–bill-tip axis (marked with a white triangle). These diagrams employ standard latitude–longitude coordinate systems, with the equator aligned vertically in the sagittal plane at 20° intervals. Conceptually, the bird’s head is positioned at the centre of a transparent sphere, with bill tips and field boundaries projected onto the surface, oriented as shown in panels (C) and (D). Colour coding indicates binocular regions (green), monocular regions (pink) and blind regions (blue). Horizontal cross-sections of visual fields, corresponding to the lines shown in panels (C) and (D), are presented for males (G) and females (H). Panels (I) and (J) display vertical sections of binocular fields when the birds focus on prey items on the ground during foraging. Panels (K) and (L) provide perspective projections of retinal field boundaries from the bird’s own viewpoint, with blind sectors highlighted in black. Finally, panel (M) illustrates the mean angular separation of retinal field margins relative to elevation in the sagittal plane for both species and sexes. Positive values denote binocular overlap, whereas negative values indicate the extent of blind zones. The coordinate framework (inset) defines the horizontal plane using elevations of −90° (behind the head), +90° (in front of the head) and 0° (directly above the head), with head orientations as shown in panels (C) and (D).

Exaggerated cranial feathers are found in the males, but not the females, of a number of species of pheasants (Galliformes, Phasianidae). Prominent examples are the two species in the genus *Chrysolophus:* golden (*C. pictus*) and Lady Amherst’s (*C. amherstiae*) pheasants [17]. Often referred to as the ‘hooded’ pheasants [18], the males of this genus, during part of their annual cycle, have ornamental feathers which produce a ruff, or ‘cape’, around their neck that can be expanded to form a fan of alternating colours, covering most of their face except for the eyes. In addition, the males have a tract of cranial ornamental feathers that protrude posteriorly and anteriorly (figure 1A). We tested the hypothesis that these cranial ornamental feathers protrude into the visual fields of the males, with the result that males, when ornamented, have a smaller extent of binocularity and a larger blind area, in comparison to the females. For comparison, we measured two closely related pheasant species (silver pheasant (*Lophura nycthemera*) and green pheasant (*Phasianus versicolor*)) which do not have the same cranial ornamentation, predicting there would be no difference in visual fields between the two sexes in these species.

## 2. Methods

### (a) Birds

The research was carried out at two private bird collections: in Arles-sur-Tech, France, and in Bedfordshire, UK. A total of four Lady Amherst’s pheasants (two males, two females), three golden pheasants (two males, one female), two silver pheasants (one male, one female) and two green pheasants (one male, one female) were studied. Husbandry information is provided in the electronic supplementary material. Previous studies have shown that visual field measurements are consistent both at the individual and population levels, indicating that a small sample size can reliably represent a species [19]. The birds were measured close to their enclosures (no more than a 5 min walk away) and were promptly returned after data collection. The maximum time any bird spent outside its home aviary was 80 minutes.

### (b) Visual field measurements

The visual field characteristics of each species were evaluated using the ophthalmoscopic reflex technique, following standardized, validated protocols detailed in prior studies [2,9,20–23]. A comprehensive explanation of this method is provided in electronic supplementary material, S1. Briefly, each pheasant was placed in a foam cradle, the body secured with Velcro tapes and the head positioned using a silicone-lined bill holder and micropore tape, ensuring comfort and ventilation. Head position matched the bird’s natural head posture. The cradle was mounted on a tripod and aligned in a visual perimeter apparatus. Corrections for bill projection and the eyes’ nodal point separation were made using calibrated photographs. The positions of the boundaries of the visual fields were measured directly behind the head in the horizontal plane and then at 10° intervals of elevation from −10° (slightly behind the head) to 110° (below the bill). See inset diagram in figure 1M for perimeter coordinate positions. The field boundaries were marked by a transition from retinal reflection to its absence (i.e. an absence of reflection). Our analysis focused primarily on the variation in binocular field topography, as well as the blind areas both anterior and posterior to the head [2,24].

### (c) Data analysis

The mean visual field data were analysed to generate vertical and horizontal cross-sections through the visual fields, as well as topographical maps of the anterior fields. Due to the limited number of species examined in this study, it was not feasible to conduct a phylogenetic generalized least squares analysis, as used in prior studies investigating the link between visual fields and foraging behaviours in Anatidae [10], Accipitridae and Cathartidae [12] and Strigidae and Tytonidae [13]. For statistical comparisons, data from Lady Amherst’s and golden pheasants were combined, resulting in a sample size of four males and three females (electronic supplementary material, table S1). Silver and green pheasants are presented descriptively (electronic supplementary material, table S2), for comparison to the *Chrysolophus* species. Silver and green pheasants were chosen due to their close relatedness to the *Chrysolophus* pheasants, last sharing an ancestor 14 and 13.5 Myr, respectively (TimeTree.org), and all four species are in the subfamily Phasianinae [17].

**Figure 2.**
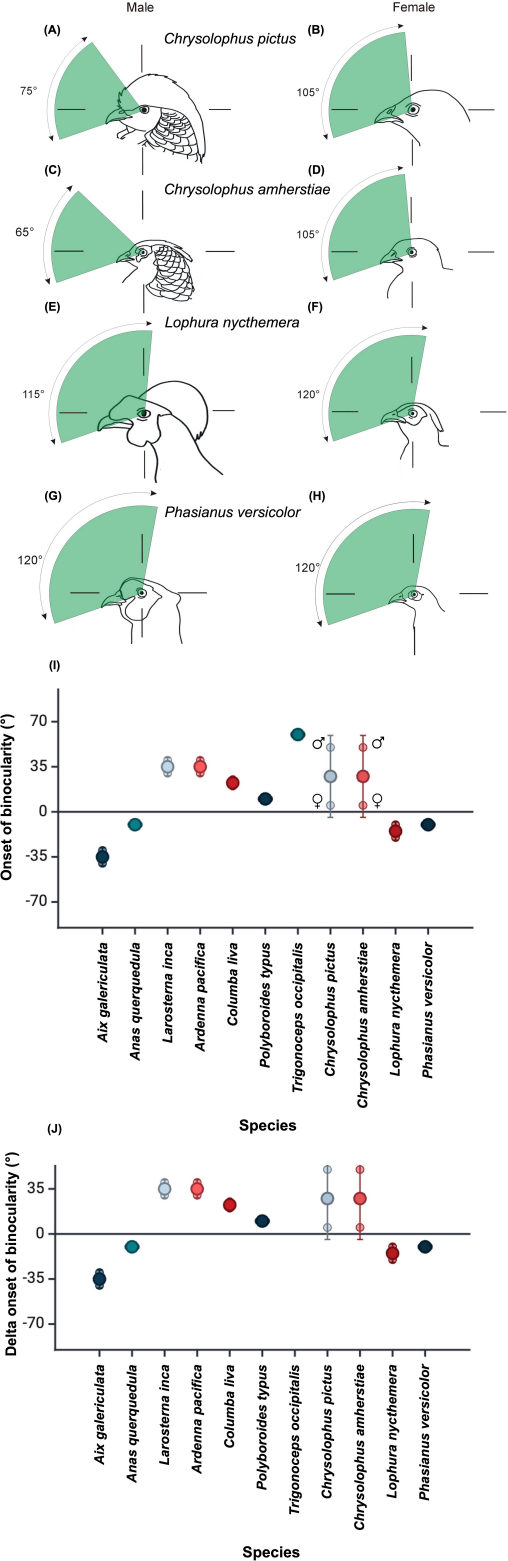
Vertical sections through the binocular fields in the median sagittal plane of the head of four pheasant species. The line drawings of the heads of the birds show them in the approximate orientations typically adopted by the species when at rest, as determined from photographs of birds held in the hand in their aviaries. The left panel shows males and right panel females of (A,B) golden (*Chrysolophus pictus*), (C,D) Lady Amherst’s (*C. amherstiae*), (E,F) silver pheasants (*Lophura nycthemera*) and (G, H) green pheasants (*Phasianus versicolor*). (I) Onset of binocularity with s.d., for 11 species (six orders). For each species, there are three data points: the mean for two individuals containing one of each sex (N.B.: many data points overlap), and the separate values for each sex. (J) Delta onset of binocularity between the two sexes. Sources of data for these comparisons (I and J) can be found in the electronic supplementary material.

**Figure 3.**
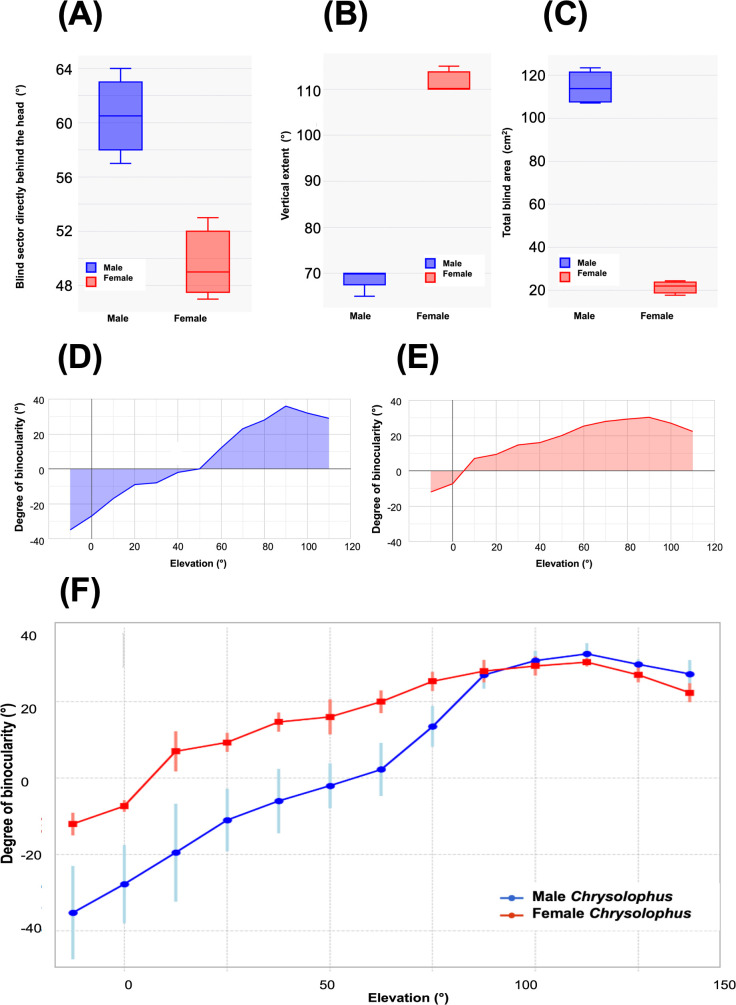
Visual fields in golden (GP) (*Chrysolophus pictus*) and Lady Amherst’s (LAP) (*C. amherstiae*) pheasants (GP^♂^*n* = 2, LAP^♂^*n* = 2; GP^♀^*n* = 1, LAP^♀^*n* = 2), with the two species combined. Data are separated by sex, not species. (A) The blind sector directly behind the head; males had significantly larger blind sectors in comparison to females. (B) The vertical extent of the binocular field; males had significantly smaller vertical extent in comparison to females. (C) Total blind area (cm^2^) measured from the blind area under the curve (D,E); males had significantly larger blind area in comparison to females. (D,E) Mean angular separation area of the retinal field margins as a function of elevation in the median sagittal plane in male (D) and female (E) *Chrysolophus* pheasants. Positive values indicate the overlap of the field margins (binocular vision), and negative values indicate the width of the blind areas (figure 1). (D) and (E) Represent the averages of the curves shown in panel (M) of figure 1. (F) The mean angular separation of the retinal field margins as a function of elevation in the median sagittal plane of *Chrysolophus* pheasants, separated by sex (males, blue; females, red). Error bars represent s.d.

All statistical analyses were performed using the statistical software R v. 4.2.2 [25], and all data are available in the electronic supplementary material. The differences in visual field characteristics between the *Chrysolophus* sexes were determined using *t*-tests with a Bonferroni correction applied for multiple testing (*n* = four tests) [26]. As such, all results are considered at the *α* = 0.01 level of significance. The following characteristics between the sexes were statistically compared: (1) vertical extent of binocularity (°); (2) maximum binocular width (°); (3) the width of the blind sector behind the head (°); and (4) total blind area (cm^2^); derived from the blind area under the curve (figures 1–3). We combined the data of golden and Lady Amherst’s pheasants by sex, to (1) increase statistical power, and (2) examine the two *Chrysolophus* pheasants collectively, given their morphological similarities. To support this approach, we first confirmed statistically that there were no significant differences between the sexes of the two species. Values are presented as mean ± s.d., unless otherwise stated.

## 3. Results

### (a) General visual field characteristics of *Chrysolophus* pheasants

Both species have a vertically long and narrow binocular field, with the visual projection of the eye–bill-tip occurring within the ventral portion of the binocular field below the point of its maximum width (figures 1 and 2; electronic supplementary material, table S1). Both species have a blind area, which starts in the dorsal quadrant of the frontal field and extends above and behind the head down to the horizontal. Maximum binocular width did not differ significantly between the sexes, when both species were combined (*t*‐test, *t*_5_ = 2.6, *p* = 0.1; female mean 30.7 ± 0.9°, male mean 33.5 ± 1.0°; figure 1G,H; electronic supplementary material, table S1). There was no overall significance in the visual fields of the males between Lady Amherst’s and golden pheasants (*t*‐test, *t*_50_ = 2.0, *p* = 0.7), or between the females (*t*‐test**,** *t*_37_ = 0.2, *p* = 0.8).

### (b) Sex differences in the visual fields of *Chrysolophus* pheasants

In addition to these general visual field characteristics found in both sexes in both species, there are significant differences between the sexes in respect of the following: (1) the binocular region has a smaller vertical extent in males than in females. Thus, from the elevation at which it could be measured below the bill, binocularity extends vertically through 75° in male golden pheasants and 105° in females (figure 1C,D), and 70° in male Lady Amherst’s pheasants and 110° in female Lady Amherst’s pheasants (electronic supplementary material, table S1, figure 2). The mean vertical extent for the two pheasant species combined was 71.2 ± 2.5° in males and 108.3 ± 2.8° in females. This difference (41.3% greater in females than males, with both species combined) was statistically significant (*t*‐test, *t*_5_ = 2.6, *p* < 0.001; figure 3B,D–F). (2) The width of the blind sector directly behind the head (elevation −90°, see figures 1M and 3A,D–F) differed significantly between the sexes when both species were combined, with females exhibiting a significantly smaller (19.6%) blind area in comparison to males (*t*‐test, *t*_5_ = 2.6, *p* < 0.001; female mean 49.7 ± 3.1°, male mean 60.5 ± 3.9°; figures 1G,H and 3A,C–F). (3) The blind region in the dorsal anterior sector of the visual field starts at an elevation between 40° and 50° in male *Chrysolophus* pheasants and between 0° and 10° in female *Chrysolophus* pheasants (figure 1K–M). The total blind area, derived from the area under the curve (figures 1M and 3D,E), was significantly larger in males than females, when both species were combined (*t*‐test, *t*_5_ = 2.6, *p* < 0.001; female mean 21.3 ± 3.4 cm^2^, male mean 114.5 ± 8.2 cm^2^; figure 3C–F). The s.d. error bars did not overlap between the sexes until 110° elevation (figure 3F).

### (c) Comparison of visual field characteristics between *Chrysolophus* pheasants and silver and green pheasants

Male and female silver pheasants had a binocular field vertical extent of 115° and 120°, respectively (figure 2), and green pheasants exhibited no difference in vertical extent between the sexes (120°; figure 2). This contrasts with the 30° and 40° difference in vertical extent between the sexes in golden and Lady Amherst’s pheasants, respectively (figures 1 and 2). The onset of binocularity was similar between the sexes in both silver and green pheasants (detected at elevations of −20° and −10° for male and female silver pheasants, −10° and −10° for male and female green pheasants, respectively; electronic supplementary material, table S2). In comparison, binocular onset in male golden and Lady Amherst’s pheasants was at an elevation 40° less than females, for both species (figures 1 and 2; electronic supplementary material, tables S1 and S2). In common with the *Chrysolophus* pheasants, maximum binocular width was similar between the sexes of both silver and green pheasants (39° and 41° for male and female silver pheasants, 45° and 49° for male and female green pheasants, respectively).

### (d) Sex difference comparisons to other species

Published visual field data where the sex of the individuals were identifiable (*n* = 7 species; six orders) were compiled, and further show that only the *Chrysolophus* pheasants exhibited a noticeable difference between the sexes in the elevation of the onset of binocularity, both in absolute terms (figure 2I) and between-sex delta onset of binocularity (figure 2F).

## 4. Discussion

This study demonstrates that a secondary sexual characteristic—exaggerated cranial feathers—can influence sexual dimorphism in visual field topography. In both golden and Lady Amherst’s pheasants, these cranial feathers reduce the extent of the binocular field and enlarge the blind area in males compared with females of the same species. While the broader implications of this finding are considered below, it is important to first note that the overall structure of the visual fields in these species is not unusual; their general features are consistent with the visual guidance of the bill during foraging.

### (a) General topography of the binocular fields

In both *Chrysolophus* species, and in the silver and green pheasants, the binocular field shows the same general characteristics; the binocular field is relatively narrow and vertically long, and the maximum width occurs above the horizontal but with the bill projecting centrally in the field below the horizontal (figure 1E,F). All of these features have been associated with the use of visual information extracted from the optic flow-field to control bill position and bill timing in foraging [8]. This correlates well with the diets of these species which include seeds, grains, berries, shoots, insects, spiders and small amphibians and reptiles [18]. The broad spans of the total visual fields in the horizontal plane in all four species and in both sexes of *Chrysolophus* (figure 1G,H) are probably associated with the detection of predators [27]. The capture and ingestion of both static and moving prey items require accurate placement of the bill and its opening, and these requirements, as in other bird species, have probably driven the maximum binocular width recorded in these species. Thus, the similarity of maximum binocular field width in these pheasants correlates well with the visual ecology of their foraging behaviour.

### (b) Sex differences in the blind areas of *Chrysolophus* pheasants

To date, visual fields have been measured in approximately 300 bird species drawn from 20 of the 44 bird orders, and 52 of the 253 bird families currently recognized [17]. However, sex differences in any parameters of visual fields have not been reported. In both *Chrysolophus* pheasant species, and in both sexes, the blind area starts in the dorsal portion of the anterior field and extends above and behind the head, reaching down to the horizontal plane. The 30° difference in the vertical extent of the blind area above the head between the sexes—with males having a significantly larger blind area—is likely to have ecological implications. The enlarged blind area would leave males generally more vulnerable to predation than females, especially when foraging with the head rotated forwards with the bill pointing towards the ground. For male pheasants, tilting the head forward by 20° to search for objects on the ground results in a large blind area projecting upwards and forwards from which no visual information can be extracted, and this is illustrated in figure 1I–L. This relative enlargement of the blind region in males may also result in higher levels of vigilance behaviour, and this would seem worthy of investigation [28].

Male *Chrysolophus* pheasants had a significantly wider blind region behind the head than females, on average *ca* 10° broader than in females (figure 1G,H). However, the overall size of the blind areas behind the head in both sexes of the *Chrysolophus* pheasants is not particularly unusual among birds. Much larger blind areas are seen in storks (Ciconiiformes) and birds of prey (Accipitriformes and Strigidae), notably the *Gyps* vultures, where blind areas up to 75° wide have been recorded [14,16,21,29,30]. The blind regions in both sexes of the *Chrysolophus* pheasants are more similar to those seen in seabirds (Charadriiformes, Procellariiformes) [24,31], cranes (Gruiformes) and bustards (Otidiformes) [21]. The important difference between all of these species and the *Chrysolophus* pheasants lies in the increased extent of the blind area in the anterior-dorsal quadrant of the visual field.

### (c) Enlarged blind area and cranial feathers in male *Chrysolophus* pheasants

The cranial feathers of male *Chrysolophus* pheasants define the limits of the enlarged blind area in the anterior-dorsal quadrant and so could be regarded as resulting in a visual impediment or ‘handicap’. The ‘handicap hypothesis’ explains how animals with apparently costly or extravagant traits can signal their fitness to potential mates, despite the apparent drawbacks of such features (*sensu* [32]). This hypothesis highlights the role of honest signalling in sexual selection [33]. Classic examples include the peacock’s large, colourful train, which makes the male more susceptible to predators, and the large antlers of male deer, which are energetically taxing and hinder escape from predators. The reduced visual field in males compared to females in these pheasants, as a result of feather ornamentation, could be viewed as a relative handicap (but see [34]).

The moulting phenology of cranial feathers in wild *Chrysolophus* species remains poorly documented, with most insights derived from anecdotal accounts in captive birds. However, it is known that males of both species typically undergo a head feather moult in September and October, following the completion of their full body moult [18]. During this time, all the head and ‘cape’ feathers are replaced, likely clearing the cranial feathers from obstructing the anterior-dorsal portion of the binocular field. This is the first known instance where a bird’s visual field probably changes during its annual cycle.

The two *Chrysolophus* pheasant species are the first documented to exhibit a sex-based difference in visual fields. Although it remains unclear whether previous research on avian visual fields has specifically sought to examine this distinction, the standard practice involves measuring both males and females within the species. If significant sexual dimorphism were present in other species, large standard errors would likely have emerged. However, prior studies have shown that individual variation within a species generally differs by less than 5° across elevation angles [9,19]. This contrasts sharply with the marked variation between sexes observed in the current study. This suggests further investigation of other species where cranial ornamentation is present may provide further evidence for sex-based differences in visual fields associated with secondary sexual characteristics. Such avian families could include Cacatuidae (cocktaoos), Paradisaeidae (birds-of-paradise), Otididae (bustards) and other members of the Phasianidae (pheasants).

## Data Availability

All data are available as electronic supplementary material. Supplementary material is available online [35].
